# Great Achievements Accomplished

**Published:** 1919-02-01

**Authors:** 


### THE DEMOBILISATION OF THE RED CROSS.

### Great Achievements Accomplished.

Like everything else in this portentous war, the activity of the Red Cross, combined with the Order of St. John of Jerusalem, has been on a gigantic scale. The combined body has built, equipped, and staffed hospitals, it has kept prisoners and captives alive by furnishing them with food and comforts, it has tended the sick, treated the wounded, cared for the convalescent, traced the missing, and filled the hungry with good things. In France, in Belgium, in Italy, at Salonika, in Palestine, in Mesopotamia, wherever there has been fighting, wherever there have been wounded, wherever there have been famished men, women, or children, suffering from the hideous ravages of the greatest war that has ever devastated the world, there the Red Cross and the Order of St. John have carried their benign activity, and there they have worked side by side with the devastators to repair the devastation.

The full tale of the achievements of these combined bodies staggers imagination. Their task requires the command of enormous funds, and the collection of this money was of itself a labour before which the most sanguine and energetic might have sat down in helpless despair; but there were willing and able helpers, who estimated correctly the charity and benevolence of the world at large as the German estimated correctly the greed and bloodthirstiness and blackguardism of his own kind. The Press, headed and inspired by the *Times*, issued an appeal to the nation and to the whole world of neutrals, and the response was not only noble and even lavish, which might have been expected, but also it was continuous, which could scarcely have been anticipated. It never ceased; it never slackened. From all the countries on earth, from every class and every calling, money poured in. America contributed in proportion to her vast wealth; remote and small communities, such as Singapore, Hong-Kong, and Fiji, supported according to their means. A perpetual stream of money, from the lordly cheque of the millionaire to the hard-earned widow's mite, poured into the coffers of the Joint War Committee. Many who had no money to give gave service, gave it freely and lavishly, and without this contribution the work of the Committee could scarcely have been carried on at all, could certainly not have been carried on to the extent and with the marvellous economy of administration with which it was in fact effected. Every one who has had to do with the collection of money for charity knows how much it costs to collect, and how large a proportion of the gross receipts is commonly absorbed in expenses of collection and administration. The oldest hands at this business will scarcely believe that a sum of fourteen millions has been collected at a cost of two-pence-halfpenny in the pound.

The expenditure of the money has been as great an achievement as its collection, and the objects on which it has been expended have been almost as various as the sources from which it has been collected. Hospitals have been built, equipped, and staffed entirely by the Joint Committee, which has not only provided the buildings, the beds, the appliances, and the drugs, but has paid the staffs, has provided them with uniforms of its own design, with distinctive buttons and badges. And this is but one branch of its multifarious activities. It not only treats the wounded and sick in its hospitals, but also provides ambulances to bring them and to take them away. It not only engages and pays its nurses, but also provides hostels for them in London where they can stay for the night when they pass through on going for their leave and on returning from it. More than this, since nurses also are subject to the ills that affect humanity, the Joint Committee provides luxurious homes at Cannes, at Le Tonguet, and elsewhere, in which nurses who have been ill or overworked may rest and recuperate before resuming their labours.

Nor is it only for the sick and wounded and for their nursing and medical attendance that the Joint Committee provides. It supplies prisoners of war with parcels of food and clothing, thus extending its life-saving activities in another direction, for without its assiduous attention and care many and many a prisoner who is now happily returned to this country would have perished of starvation, neglect and brutality at the hands of his German captors. In addition to this, the Joint Committee has rendered incalculable service in tracing thousands of missing.

More than all this, the Red Cross and the Order of St. John have complied with the very natural but very inconvenient, wishes of contributors to spend their separate funds in special ways. The British Farmers support one hospital, the Liverpool Merchants another, many single contributors and bodies of contributors have provided ambulances, which have been called after them or named by them. One munificent contributor supplied an ambulance train complete.

Now the forces of the Joint Committee are to be demobilised, but this does not necessarily mean that the staff is to be absorbed at once into the civil population. For instance, the British Farmers' Hospital is to be transferred, material and *personnel*, bodily to the Belgian Army, many of whose soldiers have already been under its treatment, and other units are being disposed in similar ways. Still, the Joint Committee, as an organisation, is winding up its affairs as far as they concern the past war, and the admirable character of its administration is illustrated by nothing more clearly than by these two very diverse facts: first, that from beginning to end there has been no complaint either of inefficiency or of excessive and muddling zeal, and, secondly, that in spite of the enormous demands upon them, the funds have been so well administered that, before the affairs of this war are wound up and the staff demobilised, it has been found practicable to cease from any further appeal for funds.

